# Selecting deep brain stimulation or infusion therapies in advanced Parkinson’s disease: an evidence-based review

**DOI:** 10.1007/s00415-012-6798-6

**Published:** 2013-01-05

**Authors:** Jens Volkmann, Alberto Albanese, Angelo Antonini, K. Ray Chaudhuri, Carl E. Clarke, Rob M. A. de Bie, Günther Deuschl, Karla Eggert, Jean-Luc Houeto, Jaime Kulisevsky, Dag Nyholm, Per Odin, Karen Østergaard, Werner Poewe, Pierre Pollak, Jose Martin Rabey, Olivier Rascol, Evzen Ruzicka, Michael Samuel, Hans Speelman, Olof Sydow, Francesc Valldeoriola, Chris van der Linden, Wolfgang Oertel

**Affiliations:** 1 Department of Neurology, University Clinic of Würzburg, Josef-Schneider-Str. 11, 97080 Würzburg, Germany; 2 Dipartimento di Neuroscienze Cliniche, Istituto Nazionale Neurologico Carlo Besta, Università Cattolica del Sacro Cuore, Milan, Italy; 3 IRCCS San Camillo, Venice, Italy; 4 Department of Neurology, King’s College Hospital, London, UK; 5 Medical and Dental School, University of Birmingham, Birmingham, UK; 6 Department of Neurology, Academic Medical Center, Amsterdam, The Netherlands; 7 Department of Neurology, Christian-Albrechts-University, Kiel, Germany; 8 Department of Neurology, Philipps-University Marburg, Marburg, Germany; 9 Service de Neurologie, CIC 802, CHU de Poitiers, Poitiers, France; 10 Movement Disorders Unit, Neurology Department, Sant Pau Hospital, Universitat Autònoma de Barcelona and CIBERNED, Barcelona, Spain; 11 Department of Neuroscience, Neurology, Uppsala University, Uppsala, Sweden; 12 Department of Neurology, Klinikum-Bremerhaven, Bremerhaven, Germany; 13 Department of Neurology, Aarhus University Hospital, Aarhus, Denmark; 14 Clinical Department of Neurology, Innsbruck Medical University, Innsbruck, Austria; 15 Service de Neurologie, Hòpitaux Universitaires de Genève, Genève, Switzerland; 16 Department of Neurology, Assaf Harofeh Medical Center, Tel Aviv University, Zerifin, Israel; 17 Departments of Clinical Pharmacology and Neurology, INSERM CIC9302 and UMR 825, University Hospital and University of Toulouse III, Toulouse, France; 18 Department of Neurology, 1st Medical Faculty, Charles University, Prague, Czech Republic; 19 Department of Neurology, University of Amsterdam, Amsterdam, The Netherlands; 20 Department of Neurology, Karolinska University Hospital, Stockholm, Sweden; 21 Neurology Services, Movement Disorders Unit, Hospital Clinic i Provincial, Barcelona, Spain; 22 Department of Neurology, AZ Sint Lucas, Ghent, Belgium

**Keywords:** Apomorphine, Deep brain stimulation, Duodenal levodopa infusion, Parkinson’s disease

## Abstract

Motor complications in Parkinson’s disease (PD) result from the short half-life and irregular plasma fluctuations of oral levodopa. When strategies of providing more continuous dopaminergic stimulation by adjusting oral medication fail, patients may be candidates for one of three device-aided therapies: deep brain stimulation (DBS), continuous subcutaneous apomorphine infusion, or continuous duodenal/jejunal levodopa/carbidopa pump infusion (DLI). These therapies differ in their invasiveness, side-effect profile, and the need for nursing care. So far, very few comparative studies have evaluated the efficacy of the three device-aided therapies for specific motor problems in advanced PD. As a result, neurologists currently lack guidance as to which therapy could be most appropriate for a particular PD patient. A group of experts knowledgeable in all three therapies reviewed the currently available literature for each treatment and identified variables of clinical relevance for choosing one of the three options such as type of motor problems, age, and cognitive and psychiatric status. For each scenario, pragmatic and (if available) evidence-based recommendations are provided as to which patients could be candidates for either DBS, DLI, or subcutaneous apomorphine.

## Background and aims

The management of Parkinson’s disease (PD) becomes challenging when motor complications (e.g., motor fluctuations including loss of medication effects such as “wearing off”, end-of-dose effect, “sudden off”, and dyskinesia) can no longer be controlled adequately by changes in oral medication. Gradual worsening of these disabling phenomena has a significant impact on daily activities and social participation, important determinants of quality of life (QoL) [1–3]. If conventional drug therapy fails, three device-aided therapies can reduce the burden of motor complications in advanced PD patients: (1) apomorphine, administered subcutaneously via daytime intermittent bolus injection or continuous pump infusion; (2) continuous duodenal/jejunal levodopa/carbidopa pump infusion (DLI), administered via gastrojejunostomy; (3) deep brain stimulation (DBS)—bilateral stimulation of the subthalamic nucleus (STN), globus pallidus (GPi) or ventral intermediate thalamic nucleus (Vim). So far, no guidelines exist concerning the decision-making regarding which therapy should be chosen for individual patients.

Therefore, a group of PD experts experienced in these therapies reviewed the literature in order to provide neurologists with an evidence-based overview of the most appropriate therapy for advanced motor symptoms in patients with PD.

## Methods

A literature search (MEDLINE, EMBASE) was conducted in May of 2009 to identify relevant studies evaluating: STN– or GPi–DBS, subcutaneous apomorphine (intermittent injections or continuous infusion), or continuous DLI. Only studies assessing the chronic use of each treatment in ≥10 patients were included; all reviews, meta-analyses, and experimental studies were excluded. Studies identified were graded according to European Federation of Neurological Societies (EFNS) Guidelines [4], from class I (highest quality) to class IV (lowest quality).

A consensus group met on June 26–27, 2009 in Marburg, Germany. All members were experts in PD treatment, with experience in at least two device-aided procedures. The impact of certain clinical parameters on the outcome and the risks of each therapy were discussed extensively. As it was generally agreed that there is currently insufficient evidence to formulate definitive conclusions and recommendations, pragmatic suggestions were formulated and discussed until consensus was reached. During the discussion process and manuscript drafting, the available evidence was updated to May 2012.

## The available evidence

Numerous DBS studies were identified, therefore only classes I–III were included. Few studies of any class were found for apomorphine and DLI, necessitating the inclusion of relevant class IV studies. Table 1 outlines key characteristics and clinical outcomes observed in the available class I and II studies. In total, 53 studies were identified for DBS (published between 2000 and 2010: six class I [5–10], four class II [11–14], 43 class III [15–57]; total number of patients, *n* = 3,291), 16 for apomorphine (1993–2012: no class I or II, six class III [15, 33, 40, 58–60], ten class IV [61–70]; *n* = 612) and 12 for DLI (1998–2012: two class I [71, 72], one class II [73], nine class IV [61, 74–81]; *n* = 439). Table 2 outlines relevant studies that evaluated the effects of each therapy on the clinical parameters determined by the consensus group, discussed in Sects. 1 and 2 below.

**Table 1:** Summary of class I and II evidence for duodenal levodopa infusion and deep brain stimulation in Parkinson’s disease patients with motor fluctuations and dyskinesia

Study	Therapy versus comparator	Study type	Patients *n*; age ± SD (range) (years); disease duration ± SD (range) (years)	Follow-up	Key study outcomes	
					Efficacy	Safety
Class I studies						
*Deep brain stimulation (subthalamic nucleus or globus pallidus)*						
Follett [5]	Bilateral STN–DBS versus bilateral GPi–DBS	Long-term results of an observer-blind RCT [7]	299; 61.9 ± 8.7 (STN), 61.8 ± 8.7 (GPi); 11.1 ± 5.0 (STN), 11.5 ± 5.4 (GPi)	24 months	No differences in efficacy between therapies. Patients undergoing STN–DBS required lower dose of dopaminergic agents versus GPi–DBS (*p* = 0.02)	Visuomotor component of processing speed declined more after STN–DBS versus GPi–DBS (*p* = 0.03) Depression worsened after STN–DBS, improved after GPi–DBS (*p* = 0.02) 335 serious AEs in 83 STN–DBS patients and 77 GPi–DBS patients; no significant between-group differences at 24 m
Okun [6]	Unilateral STN–DBS versus unilateral GPi–DBS	Double-blind RCT	45; 60 ± 8.2; 12.9 ± 3.8	7 months	No significant differences in primary outcome measures (mood, cognition) between STN–DBS and GPi–DBS in the optimal DBS state Similar motor improvement observed with STN–DBS and GPi–DBS	Adverse mood effects occurred ventrally in both targets Worsening of letter verbal fluency with STN–DBS Persistence of deterioration in verbal fluency in ‘off’ STN–DBS state suggestive of surgical rather than stimulation-induced effect
Weaver [7]	DBS (STN or GPi) versus BMT	Observer-blind RCT	255; 62.4 ± 8.9 (37–83); 12.4 ± 5.8	6 months	With STN– or GPi–DBS versus BMT, significantly greater improvements in:	Neurocognitive testing suggested small decrements in some areas of information processing with DBS versus BMT DBS associated with increased risk of serious AEs versus BMT (49 vs. 15 patients, respectively; *p* < 0.001); 99 % resolved by 6 months Rate of non-serious AEs higher in older versus younger patients, but rate of serious AEs was comparable
					‘On’ time (without troubling dyskinesia) (*p* < 0.001)	
					Quality of life (*p* < 0.001)	
					Similar benefits observed in younger and older patients	
Anderson [8]	Bilateral STN–DBS versus bilateral GPi–DBS	Extension of a double-blind RCT [8]	23; 61 ± 9 (STN), 54 ± 12 (GPi); 15.6 ± 5 (STN), 10.3 ± 2 (GPi)	12 months	With STN–DBS versus GPi–DBS:	Tendency towards more cognitive and behavioral changes with STN–DBS versus GPi–DBS, mostly mild and transient
					Tendency towards greater levodopa dose reduction	
					Tendency towards greater improvement in bradykinesia	
					Similar improvement in off-medication UPDRS motor scores	
					Similar improvement in dyskinesia	
					No improvement in on-medication function in either group	
Smeding [9]	Bilateral STN–DBS versus unilateral GPi–DBS	Substudy of an observer-blind RCT [10]	34; 59.2 ± 8.6 (STN), 62.1 ± 8.1 (GPi); 13 (3–50) (STN), 11 (7–20) (GPi)	12 months	With STN–DBS versus GPi–DBS:	One STN–DBS patient showed severe confusion and cognitive decline after surgery
					Significantly smaller improvement in two tests of executive function (stroop color word; trailmaking) at 6 months (*p* < 0.05)	
					No significant differences at 12 months	
Esselink [10]	Bilateral STN–DBS versus unilateral pallidotomy	Observer-blind RCT	34, 61 (range 55–66) (STN), 62 (range 57–68) (pallitodomy)	6 months	With STN–DBS versus pallitodomy, significantly greater improvements in:	More pallitodomy patients experienced AEs versus STN–DBS patients One major AE in each group
					Off UPDRS motor scores (*p* = 0.002)	
					On UPDRS motor (*p* = 0.02) and duration of dyskinesia (*p* = 0.004)	
					Reduction of anti-PD drugs (*p* = 0.02)	
*Continuous duodenal levodopa infusion*						
Nyholm [71]	Levodopa/carbidopa gel infusion versus conventional treatment	Observer-blind, crossover RCT	24; median 68 (51–79) (oral/infus), median 64 (50–75) (infus/oral); NS	3 + 3 weeks	With DLI versus conventional treatment, significantly greater improvements in:	AEs (e.g., dyskinesia/hyperkinesia, constipation, depression, etc.) similar for DLI versus conventional treatment
					Functional ‘on’ time (*p* < 0.01)	
					Off time (*p* < 0.01)	
					Median UPDRS score (*p* < 0.05)	
					Quality of life (*p* < 0.01)	
					Dyskinesia unchanged versus baseline	
Class II studies						
*Deep brain stimulation (subthalamic nucleus or globus pallidus)*						
Williams [12]	DBS (STN or GPi) versus BMT	Open label, RCT	366; 59 (37–79) (DBS), 59 (36–75) (BMT); 11.5 (2.0–32.2) (DBS), 11.2 (1.0–30.0) (BMT)	12 months	At 1 year, 75 DBS versus 21 BMT patients reported no waking day dyskinesia (*p* < 0.0001) and 45 DBS versus 5 BMT patients reported no off time (*p* < 0.0001) Compared with baseline, mean improvement in PDQ-39 was 5.0 points with DBS versus 0.3 points with BMT (*p* = 0.001) Difference between DBS versus BMT in mean change in PDQ-39 mobility domain was −8.9 (*p* = 0.0004), activities of daily living domain was −12.4 (*p* < 0.0001), bodily discomfort domain was −7.5 (*p* = 0.004)	In total, 19 % DBS patients had serious surgery-related AEs; there were no suicides but there was one procedure-related death Twenty patients in the DBS group and 13 in the BMT group had serious AEs related to PD and drug treatment
Witt [11]	Bilateral STN–DBS versus BMT	Open label, RCT (ancillary study to Deuschl [14])	123; 60.2 ± 7.9 (DBS), 59.4 ± 7.5 (BMT); 13.8 ± 6.3 (DBS), 14.0 ± 6.1 (BMT)	6 months	Overall, STN–DBS did not reduce cognition and affectivity With STN–DBS versus BMT:	Severe psychiatric AEs observed in ten STN–DBS patients and eight BMT patients
					No significant difference in scores for overall cognition and affectivity	
					Significantly greater reduction in anxiety (*p* < 0.0001)	
					Adverse changes in verbal fluency and performance in the Stroop test not associated with changes in psychiatric scales and did not affect improvements in QoL	
Schüpbach [13]	Bilateral STN–DBS versus BMT	Open label, RCT	20; 48.4 ± 3.3 (DBS), 48.5 ± 3.0 (BMT); 7.2 ± 1.2 (DBS), 6.4 ± 1.1 (BMT)	18 months	QoL improved to a greater extent with STN–DBS versus BMT (*p* < 0.05) After 18 months, severity of parkinsonian motor signs in medication-off conditions, levodopa-induced motor complications, and daily levodopa dose reduced with STN–DBS versus baseline and increased with BMT versus baseline	AEs were mild or transient Overall psychiatric morbidity and anxiety improved with DBS
Deuschl [14]	Bilateral STN–DBS + BMT versus BMT alone	Open label, paired RCT	156; 60.5 ± 7.4 (DBS), 60.8 ± 7.8 (BMT); NS	6 months	DBS + BMT had greater beneficial effect than BMT alone on: QoL (PDQ-39; *p* = 0.02) Severity of symptoms without medication (UPDRS-III; *p* < 0.001)	Serious AEs more common with DBS + BMT (10 events) versus BMT alone (3 events) (*p* < 0.04), but majority resolved without permanent complications Frequency of AEs higher with BMT alone versus DBS + BMT (*p* = 0.08)
*Continuous duodenal levodopa infusion*						
Nyholm [73]	Oral sustained-release levodopa versus levodopa/carbidopa gel infusion	Open label, crossover RCT	12; 61.2 ± 11.0 (39–76); NS	3 + 3 weeks	With DLI versus oral levodopa: Significantly lower average intra-individual coefficient of variation for plasma levodopa concentration (*p* < 0.01) Significantly greater increase in on time and decrease in off time and dyskinesia (*p* < 0.01)	No major complications or serious AEs with either therapy

*AEs* adverse events, *BMT* best medical therapy, except infusions, *DBS* deep brain stimulation, *NS* not specified, *QoL* quality of life, *RCT* randomized controlled trial

**Table 2:** Relevant studies that evaluated the effects of device-aided therapies on clinical parameters determined by the consensus group

Clinical parameter	Device-aided therapy	Total number of studies	Number of studies in each class [reference]				Total number of patients	Follow-up
			Class I	Class II	Class III	Class IV^a^		
Target motor symptoms of PD (severity of off-period symptoms, motor fluctuations, dyskinesia, tremor)	DBS	23	3 [5, 7, 10]	2 [13, 14]	18 [12, 15, 18, 20–22, 24, 33, 41–43, 45, 47–53, 55, 56]	–	1,940	6 months to 6 years
	Apomorphine	12	–	–	3 [15, 33, 60]	9 [61–69]	524	6 weeks to 10 years
	DLI	11	2 [71, 72]			9 [61, 74–81]	427	3 weeks to 10 years
Age and duration of disease	DBS	9	1 [7]	2 [13, 14]	6 [33, 34, 41, 43, 44, 53]	–	601	6 months to 4 years
	Apomorphine	5	–	–	1 [33]	4 [62–64, 70]	141	6 weeks to 20 months
	DLI	6	1 [71]	–	–	5 [75–79]	236	3 weeks to 11 years
Cognitive status	DBS	16	3 [6, 7, 9]	2 [11, 14]	11 [15, 16, 19, 25, 26, 31, 37, 40, 46, 54, 57]	–	1,149	6 months to 5 years
	Apomorphine	2	–	–	2 [40, 63]	–	98	12–20 months
	DLI	5	–	–	–	5 [75–79]	212	6 months to 4 years
Neuropsychiatric status	DBS	7	3 [5, 7, 9]	2 [11, 15]	2 [23, 57]	–	927	6 months to 5 years
	Apomorphine	4	–	–	2 [58, 59]	2 [63, 68]	161	12 months to 3 years
	DLI	5	–	–	–	5 [75–79]	212	6 months to 4 years
Medical co-morbidities	DBS	3	1 [7]	1 [14]	1 [33]	–	436	6–12 months
	Apomorphine	2	–	–	1 [33]	1 [63]	107	12–20 months
	DLI	5	–	–	–	5 [75–79]	212	6 months to 4 years
Non-motor symptoms	DBS	1	–	1 [14]	–	–	156	6 months
	Apomorphine	None	–	–	–	–	–	–
	DLI	2	–	–	–	2 [76, 78]	35	6–12 months
Dysarthria	DBS	4	1 [7]	1 [11]	2 [34, 47]	–	449	6 months to 5 years
	Apomorphine	None	–	–	–	–	–	–
	DLI	None	–	–	–	–	–	–
Gait/balance problems	DBS	7	1 [7]	–	6 [21, 29, 34, 36, 39, 47]	–	470	6 months to 5 years
	Apomorphine	1	–	–	–	1 [63]	82	20 months
	DLI	1	–	–	–	1 [75]	91	Up to 48 months

*DBS* deep brain stimulation, *DLI* duodenal levodopa infusion

^a^No class IV studies were included for DBS

The current evidence best supports DBS, with more well-designed, i.e., prospective, randomized controlled trials (RCTs) compared to infusion therapies. In addition, class I and II studies directly compared DBS with best medical (pharmacological) treatment (BMT), which sometimes included apomorphine (e.g., [12]). In contrast, few prospective RCTs exist for DLI and none for apomorphine; most were non-randomized, uncontrolled, open-label, retrospective, generally small and with short follow-up, or were case studies. Nevertheless, these data provide clinically useful information. Five studies directly compared any of the three therapies [15, 33, 40, 61, 74], only one compared all three [61]. Even more importantly, treatment decisions in a clinical setting are often influenced by individual factors that may represent exclusion criteria in controlled studies (e.g., older age, neuropsychiatric comorbidity, frailty), exemplifying the need to include naturalistic studies with scenarios that are excluded from RCTs.

## Managing the target motor symptoms of PD

### Is any device-aided therapy preferable regarding efficacy in managing target motor symptoms of PD?

#### Severity of off-period symptoms

The impact of DBS on off-period symptoms is typically analyzed in an artificial situation, which does not reflect the clinical practice of treating patients continuously with an optimized combination of stimulation and medication. The observed improvement in off-period symptoms (UPDRS motor score) induced by STN–DBS ranged between 30 and 60 % in larger trials. There was less and lower quality data on the impact of GPi–DBS, but it seemed to be around 30 %. Although apomorphine infusion or DLI have never been formally assessed for their impact on off-period symptoms in a setting comparable to DBS studies (they provide continuous drug, so a levodopa challenge test would make little sense in their clinical evaluation), one would expect them to have an at least equivalent, if not short-term superior effect, if given in appropriate dosages.

#### Motor fluctuations

*DBS* (*STN and GPi*) The reduction in daily off-time with DBS was variable and ranged between 30 and 100 % (median 68 %). Each study demonstrated significant reductions in off-time. Similarly, increase in on-time without dyskinesia ranged from 47 to 138 % (median 71 %). *Apomorphine* Data specifically addressing fluctuations are limited: overall reduction in off-time varied from 25 to 80 %, with lower values being reported more often (median 44 %). However, results were rarely recorded on a daily chart per hour, making it difficult to assess the results’ true value. Off-time reduction was restricted to the daytime; nocturnal akinetic periods were not addressed. An increase in on-time without dyskinesia was rarely reported, ranging from 8 to 85 % (median 40 %). *DLI* Off-periods were reduced by 40–80 % (daytime off only as DLI was mostly discontinued at night). One study reported increase in on-time without dyskinesia (88 %).

#### Dyskinesia

*DBS* (*STN and GPi*) Dyskinesia reduction ranged from 70 to 100 %. DBS also reduced dyskinesia severity by up to 83 %. Dyskinesia benefits were consistently reported. In STN–DBS, dyskinesia alleviation was related to the reduction of dopaminergic medication, not directly attributable to neurostimulation itself. *Apomorphine* Apomorphine had a variable effect on dyskinesia, ranging from no reduction to 70 % reduction (greatest reductions in older studies). Dyskinesia benefits depended on oral levodopa withdrawal and mostly referred to patients on oral levodopa monotherapy before infusion. *DLI* Dyskinesia time was reduced by 60–90 %. This was not related to total daily levodopa dosage reductions, but to the more continuous dose distribution.

#### Tremor

*DBS* (*STN and GPi*) DBS had a beneficial effect on tremor (and bradykinesia and rigidity, also cardinal motor PD symptoms). *Apomorphine and DLI* The effects of either therapy on tremor were not clearly addressed.

#### Conclusions

In general, more evidence exists for the efficacy of DBS on motor fluctuations and dyskinesia versus DLI and apomorphine. Consistent results with DBS indicated its efficacy at reducing off-period motor symptoms and increasing on-time without dyskinesia. Apomorphine is likely effective at reducing daytime motor fluctuations and has a variable effect on dyskinesia, but existing evidence is too poor to permit firm conclusions. Off-time reduction with DLI is limited to the daytime; some nighttime effects have been reported [82]. Although sometimes practiced, there are concerns about the safety of 24-h dopaminergic infusion therapy and uncertainty regarding nighttime dose adjustments required. Preliminary data suggest that DLI has a strong beneficial effect on dyskinesia [72], but lack of formal evidence precludes firm conclusions. For disabling tremor, DBS can be effective even if tremor was unresponsive to levodopa or other oral dopaminergic drugs.

### What patient-related factors may influence the choice of therapy?

#### Age and duration of disease

Does the patient’s age or duration of PD prior to treatment affect the outcome of therapy? If so, can it be used as a predictor of response?

*DBS* Younger age predicts a more favorable response to bilateral STN–DBS regarding motor and QoL improvement. There are concerns that progression of axial motor signs and emerging dementia may counteract improved activities of daily living (ADL) after STN–DBS in older patients (>70 years). However, in a study where 25 % of patients were >70 years [7], there was comparable benefit in younger and older patients regarding on-time without dyskinesia and off-period UPDRS motor scores. There was a higher risk of non-serious adverse events (AEs) in older patients, but no difference between older and younger patients in the rate or type of serious AEs [7]. A large series did not observe a greater risk of bleeding in elderly patients [83].

Regarding disease duration, reliable long-term results (up to 8 years) have been observed in patients with PD for a mean of 15 years before surgery, but the risk of evolving dementia or gait problems appears higher with a longer disease duration. However, the “window of opportunity” for STN–DBS may open earlier, when fluctuations and dyskinesia emerge and start to impact on ADL in younger patients [13].

*Apomorphine* Apomorphine was effective in older patients (up to 85 years) with a long disease duration. No relationship between age and disease duration on the outcome of treatment was observed. No studies stratified AEs by age.

*DLI* DLI can be effective in patients of all ages and with a long disease duration. DLI appears an effective last-line therapy for PD motor complications, with suggestions to prefer it over other device-assisted therapies in older, frail patients because of better tolerability.

#### Conclusions

While STN–DBS can confer improvements in motor symptoms in older patients (>70 years), it may provide greater benefits in younger patients regarding ADL and QoL, with a seemingly lower risk of AEs. STN–DBS should not be considered a treatment of last resort as better results might be obtained in younger patients with a shorter duration of motor complications [84]. The durability of the treatment effect, which can be counteracted by the evolution of axial motor symptoms and cognitive decline [85], should be discussed with older surgical candidates. The efficacy of apomorphine on motor symptoms does not seem to depend on age or disease duration, but there are insufficient and contradictory data [70] to conclude on safety in older patients, in particular the risk of psychosis and confusional states. There is no evidence of an age-related decline with DLI, which seems to be well tolerated, even in older patients with very advanced PD including some cognitive decline.

#### Cognitive and neuropsychiatric status

##### Cognitive status

What effect does each therapy have on the patient’s cognitive status? Can this be used to recommend a treatment?

*DBS* No safety data exist in patients with co-existing dementia and active psychiatric symptoms at baseline, as they are usually excluded from DBS studies. In patients with normal neuropsychological testing before surgery, some frontal executive function measures (e.g., verbal fluency) decreased with DBS, but no changes in global cognitive scores were found. No significant differences were seen with stimulation ‘on’ or ‘off’ [11, 57], so the observed effects on frontal executive function are less likely caused by neurostimulation per se, but may result from surgical aspects (e.g., microlesional effect at the STN target or the trajectories of the electrodes through the frontal white or deep gray matter). Notably, small but significant deterioration in frontal executive scores did not affect daily functions or QoL. *Apomorphine* One study showed no cognitive changes, while the other noted a trend towards impaired cognitive function after apomorphine challenge, with a confused state in 17 % of patients (only half the patients completed the study). *DLI* In general, no definite conclusions could be made due to lack of study data.

#### Conclusions

STN–DBS seems cognitively safe in patients with normal age-related cognitive testing at the time of DBS. However, special care should be taken in those on a clinical course suggestive of imminent cognitive decline. It is not known whether decrements in frontal-executive functions caused by STN–DBS could aggravate natural PD-related cognitive dysfunction in the long term. Clinically recognized dementia (DSM IV) is a contraindication for DBS. Baseline characteristics (e.g., age at onset, presence of axial ‘on’ symptoms) may individually predict postoperative cognitive decline [86], and should be weighed against possible motor benefits. Few or inconclusive data exist on the cognitive safety of apomorphine or DLI but, except for the risk of acute confusional states with dopaminergic therapy, they should not impact on the natural evolution of dementia in PD.

#### Neuropsychiatric status

Does the patient’s neuropsychiatric status before treatment affect outcomes? Can any therapy be recommended in patients with pre-existing neuropsychiatric problems?

*DBS* On average, depressive mood ratings improved after STN–DBS. However, the risk of aggravation increased in patients diagnosed with depression at baseline. Suicide risk increased within the first year after STN–DBS, along with other impulsive behavioral disorders, but returned to baseline after 3 years. Anxiety improved in most patients. Apathy improved with acute ‘on’/‘off’ stimulation, but 12–25 % of chronic STN–DBS patients developed apathy after extensive reduction of dopaminergic medication. Conflicting data exist on the effect of STN–DBS in patients with impulse control disorders or dopa dysregulation syndrome, with aggravation in some and marked improvements in others, if dopaminergic medication withdrawal was tolerated. Most neuropsychiatric problems were generally reported during the adjustment period of medication and stimulation and tended to disappear within the first 6 months.

*Apomorphine* There was a moderate improvement in mood and anxiety, but frequent induction or aggravation of visual hallucinations, confusional states, hypersexuality and paranoid psychosis. A 24-h infusion is considered unsafe because of the risk of exacerbating psychiatric complications. *DLI* Few neuropsychiatric AEs were observed, with some improvements in psychiatric symptoms (e.g., depression, anxiety, delusions, hallucinations) in patients with mild–moderate cognitive impairment and previous psychosis. The most likely reason is the change from oral antiparkinsonian polypharmacy to levodopa monotherapy.

#### Conclusions

For STN–DBS, strong evidence supports a favorable impact on mild–moderate depression and anxiety after 6–12 months, while weaker evidence suggests possible deleterious effects on apathy, psychosis, impulsivity and emotional processing. Conflicting data exist regarding effects of STN–DBS in patients with impulse control disorders or dopa dysregulation syndrome. Favorable outcomes may require withdrawal of dopaminergic medication. Ongoing psychotic or severe depressive episodes (with or without suicidal ideation) are DBS contraindications, but may be treated and DBS performed after remission. Fewer reports exist of neuropsychiatric complications with GPi–DBS, possibly reflecting publication bias.

Based on limited evidence, apomorphine may improve mood and anxiety but is associated with a risk of psychosis, confusion and disinhibited behavior. Clinical experience suggests caution in patients with impulse control disorders. The available data for DLI do not allow firm conclusions on neuropsychiatric safety, but open label data suggest it may be best tolerated of all three options in patients with a history of psychosis. No evidence exists to comment on ongoing psychosis or impulse control disorders. It is recommended that neuropsychiatric assessment is carried out before any device-aided therapy and that patients with previous psychiatric history receive post-treatment neuropsychiatric surveillance.

#### Medical co-morbidities

Do any medical co-morbidities preclude a particular treatment?

Little evidence relates to medical co-morbidities, mainly because studies excluded affected patients. *DBS* Medical contraindications for DBS apply in general for awake stereotactic neurosurgery. Severe brain atrophy or lesions interfering with trajectory planning are normally considered a surgical contraindication. Anticoagulation or cardiac devices are not strict contraindications, but complicate surgery. *Apomorphine* Diabetes mellitus, if the patient has wound healing, cellulitis or skin problems, may be problematic. *DLI* Previous abdominal surgery may not allow the placing of a gastrojejunostomy and constitutes a contraindication. Inflammatory demyelinating polyneuropathy is a possible severe AE, but the impact on preexisting polyneuropathy has not been established. The weight of the pump may be a relative contraindication or burden in frail patients. Patients should be advised about their individual risks and counseled as to whether they outweigh the expected benefits of any device-aided therapy.

#### Non-motor symptoms

What effect does each therapy have on non-motor symptoms (NMS) (e.g., sleep problems, pain, loss of energy, etc.), which many patients regard as having a greater impact than motor disorders [87]? Is it possible to choose a therapy according to the patient’s NMS?

Few studies have assessed the impact on NMS. DBS provides a 24-h effect; a beneficial effect on sleep is indirectly supported by significantly increased sleep duration versus BMT. DLI significantly improved several NMS domains, i.e. cardiovascular, sleep/fatigue, attention/memory, gastrointestinal, urinary, miscellaneous (including pain and dribbling). Because few studies have assessed these factors, NMS should not be a decisive reason for recommending a therapy.

#### Presence of dysarthria

Will any treatment have an impact on speech (dysarthria) with long-term use?

*DBS* Generally, DBS did not or only transiently improve off-period scores of speech or swallowing. Moreover, dysarthria was the most frequent non-serious AE. *Apomorphine and DLI* No data exist, but clinical observation suggests they are not likely to increase the risk of dysarthria.

#### Conclusions

STN–DBS may cause mild-moderate impairments in speech (up to 10 % of patients), whereas any mild beneficial effects do not seem to last beyond 12 months. Impairments are not always reversible or solved by adjusting stimulation parameters. The causes of dysarthria after DBS are multifactorial, including unmasking of PD-related speech problems with excessive drug withdrawal, stimulation-induced speech problems by inadvertent current spread to the internal capsule, and progression of axial motor symptoms in the long term. DBS is not recommended for patients with preexisting significant speech or swallowing difficulties. Speech is less likely to worsen with apomorphine or DLI, although no supporting published data exist.

#### Presence of gait and balance problems

What effect does each therapy have on gait, instability and the risk of falls?

Note that some studies have evaluated gait kinematics, but are not included because we aim to discuss clinical usefulness (i.e. gait in a daily setting). *DBS* Falls or gait disturbance have been reported as AEs of STN–DBS. This contrasts with significant short- and long-term improvements in off-period postural instability and gait disorder (PIGD) UPDRS subscores, as well as significant improvements in freezing of gait, gait parameters and balance. A worsening in on-period axial subscores was reported in patients >70 years, especially those with preoperative gait difficulties [44]. *Apomorphine* A significant improvement in gait imbalance has been demonstrated (one study) [63]. *DLI* DLI improved gait disorders (freezing, festination, postural instability) in almost two-thirds of patients in one study [75].

#### Conclusions

Overall, significant gait and balance improvements have been demonstrated after STN–DBS. The benefits may be greater in younger patients, closely linked to the levodopa-responsiveness of axial motor symptoms before surgery. An increased risk of falling has been reported, but it is unclear whether this relates to detrimental effects of DBS per se, to the transitional period of optimizing the interplay of DBS and medication following surgery, or paradoxically to an increased mobility in patients with preexisting postural instability. Physicians need to review the risk of falling with patients before recommending DBS. Any beneficial effects of STN–DBS on gait, posture and postural stability may diminish with the natural disease progression. STN–DBS may not match the benefits of levodopa on axial symptoms in older patients, thus leading to increased gait or balance problems with postoperative withdrawal of dopaminergic drug. Patients with levodopa-resistant postural problems before surgery are at particular risk of falls after DBS. Apomorphine and DLI may have positive effects on levodopa-sensitive gait and balance problems or on dyskinesia-related problems, but only weak supporting evidence exist.

## Recommendations when advising patients regarding treatment

Managing advanced PD is complex. Treatment needs to consider motor and non-motor symptoms as well as several other individual factors, requiring a tailored approach for each patient. Currently, no direct comparative data exist to support the use of one device-aided therapy over another. It is doubtful this evidence will be generated due to the complexity and the lack of industry drive to design trials of direct comparison.

Each therapy has generated efficacy data for off motor symptoms, on–off fluctuations and dyskinesia, although the level of evidence is currently highest for DBS. However, in day-to-day clinical practice, therapeutic decisions often need to be made in patients who would not fit the strict inclusion/exclusion criteria of clinical trials; other factors such as the severity of cognitive, psychiatric, speech, balance and general medical conditions also require scrutiny. A multidisciplinary approach towards evaluating the contribution of these factors on impaired QoL is highly recommended. A useful stepwise guide would initially involve a careful workup to address the presence of disabling motor fluctuations, dyskinesia and tremor, levodopa responsiveness, general medical condition and cognitive and neuropsychiatric status. Determination of absolute and relative contraindications should follow, as some patients will only be suitable for a single therapy while others will have greater choice. If they are eligible for several therapies, an individual risk–benefit assessment should then address the key question, which therapy is most likely to restore daily functions and QoL? Patient preference forms a significant part of the decision-making process, and identifies: the PD aspects that have the most impact on QoL; lifestyle limitations and personalized social stigma due to a device’s visibility; a patient’s ability and desire to comply with device maintenance for long-term clinical benefit. It is important that neurologists discuss QoL and lifestyle needs with patients and caregivers, and provide advice regarding the potential impact of each therapy on their lives. Full practical therapeutic details should be provided, and patients should be aware of the frequency of follow-up that is essential for all therapies and the amount of daily nursing care required for apomorphine and DLI. It is important that patients realize that each therapy is reversible, so that if one becomes unsuitable, they have the option of trying another. Reimbursement issues may have to be considered in some countries.

Communicating this level of information is complex, requiring experience by a neurologist with a good understanding of all therapies, including the advantages, possible disadvantages and practical problems, taking into account limited comparative evidence. Although it can be time consuming, it is vital to allow patients and caregivers to make an informed decision as to the most appropriate therapy to meet their specific requirements.
